# Is selective nasopharyngeal brain cooling detrimental to neuroprotection?

**DOI:** 10.1186/cc14505

**Published:** 2015-03-16

**Authors:** M Kumar, L Johnson, A Goldberg, M Kashiouris, L Keenan, A Rabinstein

**Affiliations:** 1 Mayo Clinic, Rochester, MN, USA

## Introduction

Clinical outcomes vary depending on the method used to induce therapeutic hypothermia following stroke or cardiac arrest. In swine, we tested the hypothesis that selective nasopharyngeal brain cooling (SNBC), in contrast to systemic hypothermia, adversely impacts cerebral perfusion.

## Methods

In two groups of animals (34 to 35 kg), blood flow in the right middle cerebral artery (MCA) was measured using transcranial Doppler (TCD). In group 1, SNBC was initiated using perfluorohexane aerosol (1 ml/kg/minute) and oxygen (1 l/kg/minute) through a nasopharyngeal catheter atomizer. In group 2, the animals were body surface cooled using water-circulating blankets (4°C). In both groups, brain temperature, intracranial pressure (ICP), core temperatures and vital signs were continuously recorded. Cooling was terminated once the brain reached 32°C and the animals were allowed to passively rewarm.

## Results

In the SNBC group, the brain target temperature was reached in 54 ± 11 minutes. The mean ICP dropped precipitously to a nadir of -15 mmHg. TCD showed significant vasospasm in the MCA, compared with the internal carotid artery (ICA), during the entire cooling phase (Table 1). Upon termination of cooling, the brain temperature spontaneously rewarmed to core temperature in 13 ± 4 minutes. Rewarming was associated with hyperemia and elevation of ICP. In group 2, there was no cerebral vasospasm or hyperemia during cooling and rewarming respectively.

**Table 1:** Cerebral vasospasm during SNBC.

Intervention	MCA flow velocity (cm/second)	ICA flow velocity (cm/second)	Lindegaard ratio	Pulsatility ratio	ICP (mmHg)
	62	41	1.51	0.77	13
SNBC cooling	128	52	2.46	0.48	-15
SNBC rewarming	96	44	1.74	1.12	21

## Conclusion

SNBC is associated with significant vasospasm of the MCA. In addition, spontaneous and rapid rewarming of the brain, vasodilation, rapid reperfusion, and rebound elevation of ICP, all occurring minutes after termination of SNBC, are likely to be detrimental to an already ischemic brain.

